# Correlating Multimodal Physical Sensor Information with Biological Analysis in Ultra Endurance Cycling

**DOI:** 10.3390/s100807216

**Published:** 2010-07-30

**Authors:** Gregory C. May, Aiden R. Doherty, Alan F. Smeaton, Giles D. Warrington

**Affiliations:** Centre for Sensor Web Technologies Dublin City University, Glasnevin, Dublin 9, Ireland; E-Mails: gregory.c.may@gmail.com (G.C.M.), alan.smeaton@dcu.ie (A.F.S.), giles.warrington@dcu.ie (G.D.W.)

**Keywords:** ultra-endurance cycling, body sensors, cycling event detection, estimating energy expenditure

## Abstract

The sporting domain has traditionally been used as a testing ground for new technologies which subsequently make their way into the public domain. This includes sensors. In this article a range of physical and biological sensors deployed in a 64 hour ultra-endurance non-stop cycling race are described. A novel algorithm to estimate the energy expenditure while cycling and resting during the event are outlined. Initial analysis in this noisy domain of “sensors in the field” are very encouraging and represent a first with respect to cycling.

## 1. Introduction

Over the past 30 years ultra endurance sporting events have undergone a major rise in popularity, with an increasing number of ultra endurance races occurring across a variety of sports [1–4] An ultra endurance event is commonly classified as any event over 6 hours in duration [3]. Traditionally prolonged endurance events such as the modern marathon distance (42.2 km) would have been considered an ultra endurance event. However, with the current men 128s World Record time hovering just above two hours (02:03:59, Haile Gebrselassie, Berlin 2008) this event although prolonged in nature, by the strictest definition, does not fall under the classification of ultra endurance. In contrast, the original marathon that Pheidippides is said to have run from Athens to Sparta and back, covering a distance of approximately 240 km, is closer both in distance and duration to the modern definition of ultra endurance events. Events such as ultra-marathons which are typically performed over distances ranging from 50 to 100 km or more are now becoming more commonplace and are attracting an increasing number of participants each year [3]. These events now span a wide variety of sports including; running, cycling, swimming, sailing, and triathlon. More famous events among others include the Trans Alps (running and cycling), the Trans Rockies (running and cycling), the Marathon De Sable (running) and the Devizes to Westminster race (kayaking).

During ultra-endurance races the human body is engaged in prolonged bouts of relatively intense exercise. The longer the event the more important it becomes to know how the athlete will be affected by the exercise itself. This necessitates investigation of the effects on physiological function as well as the importance of recovery post-event. The impact of ultra-endurance exercise has been shown to effect a wide range of factors including sleep deprivation, muscle damage [5], metabolic changes [6], physiological fatigue [7], and reduction in cognitive capacity [7]. By increasing the understanding of the physical and cognitive demands of participation in an ultra-endurance event it is possible to tailor training and preparation in order to minimise the effects of fatigue and optimise performance. The recent commercial availability of portable power based measuring technologies in cycling has led to a similar revolution in training and racing as first experienced with low cost portable heart rate monitors [8]. Athletes and coaches are now able to gather data, both on and offline, of performance during cycling bouts in the real world.

To date there is a dearth of scientific information available investigating the demands of ultra endurance cycling. Most are either single subject case studies, or on small groups of solo participants [7,9]. Few studies were found relating to larger sample groups. In one study data was collected on 36 participants after a 24 hour event, however this contained no metabolic data [4]. Due to the nature of a multi day ultra endurance race, small participation rates, and the logistics of frequent sampling during continual 24–72 hour racing, simultaneous evaluation of a large number of participants can prove problematic. This is further confounded by a high attrition rate during these events also makes it harder to guarantee a large sample size so selective subject choice can play a part within the group.

The purpose of this article is to investigate a broad range of physical and biological sensing devices, and intelligent processing of the data gathered by them, which may lead to a better understanding of the physiological and psychological demands of a multiday ultra-endurance competitive cycle events. This article is focused on the use case of trained cyclists in competitive racing and how the range of sensors we use, and the intelligent processing techniques of the data from them, assist in understanding the various physiological demands of ultra endurance cycling, e.g., to understand the athlete capacity to exercise during the event, and their subsequent recovery. An outline will be given of the sensors involved in this study, why they were used, the data gathered and how it was integrated and analysed. Also shown are preliminary results from the overall analysis.

The remainder of this article is organised as follows: in Section 2 an overview of ultra-endurance events is provided, why they are interesting, the demands they place on athletes and the challenge they provide for sensors. Subsection 2.1 provides an overview of the Race Around Ireland cycling event which was the sporting event used for the analysis carried out in this article. Section 3 details some of the physical conditioning and strategy of the participant athletes for this event. Section 4 gives a description of the array of physical sensors and biological markers used in monitoring activity levels throughout the cycling event. Section 5 provides an overview of the data processing techniques used to infer the energy expenditure of the athletes in the Race Around Ireland (RAI). Section 6 details the results gathered, which we discuss in further detail in Section 7. Finally in Section 8 four conclusions are presented and recommendations for further research are given.

## 2. Ultra Endurance Events

Historically, different areas within sports science have defined fatigue in various manners and it can be an area of great debate, and not an easy variable to measure absolutely. Therefore, no clear-cut definition of fatigue has been given in the literature. However it is generally agreed that fatigue is both a physiological and psychological experience. From a psychological perspective fatigue is defined as a sensation of tiredness that impairs both capability and willingness to perform a task [10]. Ultra endurance events are well suited domains towards investigating fatigue in athletes.

Ultra endurance exercise has been defined as *“. . . exercise performed at an intensity of 50–70% VO*_2_*max [11] . . . ”*, with durations greater than 4 hours [12], and *“. . . events that exceed 6 hours in duration . . . ”* [3]. However the boundary level of “ultra” racing is constantly being re-evaluated as the capabilities of human performance are constantly being expanded. However in practice an event of sub-maximal intensity (relative to non-ultra events of the same sport) over extended duration and distance, is defined as an ultra endurance event. With an expected duration of 96 hours for completion of the event over a distance of approximately 2,170 km, it is clear that the RAI constitutes an ultra-cycling event.

Endurance cycling requires the ability to generate a large mechanical force (power) over a prolonged period of time while resisting fatigue. One of the most significant predictors of the cyclist’s capability to meet the physical demands of the sport is the ability to minimise the impact of fatigue and effectively recover between bouts of exercise. In contrast to traditional cycling events such as road cycling and mountain biking, there are no normative data available relating to the anthropometric and physiological characteristics of ultra endurance cyclists. This is partially due to the relative infancy of the sport and that logistically it is not feasible to study a large population during one event.

Of the limited data available, studies carried out on races such as Race Across America (RAAM) [13] and the Race Across the Alps (RAA) [14] it has been shown that the competitors studied had similar anthropometric variables to trained road cyclists as reported in the literature [15]. Differences are seen in the duration of training and distance covered year to year, however this is to be expected due to the nature of ultra-marathon events and their extended duration. The most interesting difference is in the age of participants, where reported means show similar ages, the standard deviation shows a skewing towards older athletes competing in these ultra events [2,14,16]. This stands to reason, as maturing athletes tend to experience a fall in maximal power output whilst still being able to exercise at a relatively high intensity for extended time periods using aerobic energy sources [6].

### 2.1. The Race Around Ireland: A Team Relay Cycle Race

The RAI is an ultra endurance cycling race sanctioned by the Ultra Marathon Cycling Association (UMCA) and is one of the few races that are part of the UMCA World Cup. The event is a time trial where solo and team participants aim to complete a 2,170 km circuit in as little time as possible. Solo participants have a maximum allotted time of 120 hours, whereas teams have only 96 hours in which to complete the race. Competing cyclists are continually followed by a support car to indicate position on the road to other road users, and to allow for illumination of the rider during night time hours, 7 pm–7 am. With the follow vehicle it was possible to mount video equipment to monitor the participants at all times. This study investigated a 4 person team who had already entered the race and subsequently agreed to take part in the study.

During the event solo cyclists aim to cycle for the longest period of time possible before taking rest, in essence any time spent not cycling is time that they are losing to a competitor. The race category for a four person team however allows for different tactics to be undertaken. As only one cyclist is allowed to be actively cycling at any given time (UMCA 2009) teams have the ability to rotate riders and rest team members during the event. The strategy adopted by the four man team studied was to have two pairs of cyclists rotate the exercise periods. As two cyclists were recovering in a support van several hours ride away, two members were actively rotating cycling efforts. These rotations occurred every 20–30 minutes depending on terrain, weather, and available change-over points. This rotation allowed the subject who was actively cycling to ride at a higher intensity than they would be capable of sustaining for an extended period of time (greater than 30 minutes). At this intensity the participant could recover during the rotation period while the second cyclist was riding. However, extended exercise at this intensity would result in an inability to recover for subsequent exercise bouts due to working at a higher capacity than they could physiologically endure, thus resulting in fatigue. After an allotted period of time (4 hours during daytime, 6 hours at night) the pairs of cyclists rotated, allowing for a longer period of recovery and refuelling. During more flat stages with fewer gradient changes certain participants were selected who could maintain a constant high power output, however when the gradient increased lighter participants with higher power to weight ratios would be utilised.

## 3. Pre-Race Preparation of the Athletes

Participants were recruited from a team already entered into the RAI. All members of the four-man team came from an extensive racing and training background (road racing, triathlon and adventure racing) and they were considered well trained endurance cyclists. After ethical approval had been obtained to carry out this study, the participants underwent a detailed baseline physiological assessment. Aerobic fitness was confirmed from initial physiological testing and showed that they fell within reported means for well trained cyclists of their category, and age [15] (see Table 1).

Due to the relay nature of the RAI, a race strategy needed to be developed that would utilise the team’s strengths. Exercise testing was performed in the human performance laboratories at Dublin City University in order to assess the participants level of physical fitness. The participants’ initial visit involved a maximal incremental exercise test performed on a calibrated cycle ergometer (Velotron Dynafit Pro, Racermate, US). This ergometer allowed for the manipulation and measurement of: mechanical force and torque developed within the drive-train; cadence (pedal turnover rate); heart rate (wireless chest mounted transmitter); velocity; and distance covered.

Participants initially cycled at a resistance of 100W which increased in 50 W stages every 3 minutes until failure. Resistance was created by an electromagnetic load generator which allowed a variation in resistance through a PC program. In the last minute of each stage the following parameters were recorded: heart rate via a Polar heart rate monitor (Polar, Finland), blood lactate using a Lactate Pro hand held lactate analyzer (Arkray, Japan), inspired and expired oxygen, carbon dioxide, respiration rate and respiration volume in real time via a metabolic cart (Innocor, Denmark).

Physiological data was also captured utilising oxygen and carbon dioxide sensors, breath-by breath-respiration and volume monitoring via a metabolic cart. These were used in pre- and post-race laboratory environment trials to assess participants’ physiological function and capacity to exercise. Participants also provided venous blood draws and saliva samples during the entire study for post-race analysis of metabolic stress. A number of psychological questionnaires and test were also administered before, during and post race in order to assess mood state and cognitive function. Additionally a series of pre- and post-race assessments on the 4 participants carried out over 1 month post-event. This array of sensors allowed a better understanding of the physical condition of each participant, from their pre-race level, during various stages through the race, to their post-race recovery.

### 3.1. Strategy Formation for Cycle Race

Once the baseline testing had been completed it became possible to develop a training strategy for the team, and ultimately a pacing strategy for individual participants for the race itself. In order to further assess the demands of the race, and as there was no reported background information on the race as it was the first year, an examination of the GPS track of the course was undertaken in order to assess the route. On inspection it was found that there was relatively more climbing than descending within the race kilometre for kilometre. This necessitated developing a performance test that mimicked the terrain of the course, thus providing an insight into how the participants would perform during repeated bouts of short duration high intensity cycling. Subsequent recovery was not investigated following the simulated time trial as it was outside the scope of this study, but would have been of benefit to race strategies.

**Figure 1.**
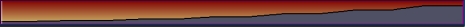
Pre-Race Trial Course Gradient.

During the simulated time trials, participants were given 20 minutes to cover as much distance as possible on the allocated course. The course was divided into kilometre long sections of increasing gradient, interspaced by a flat section, (see Figure 1). They were instructed to pace themselves, give a best effort performance, and to treat it as a race effort. Data for power output, speed, cadence, distance and heart rate were continually measured during the 20 minute effort from the ergometer itself and recorded to a PC for later analysis, Table 2. This also allowed generation of a value for calorific expenditure during the performance test. This reflects the amount of energy that this effort “cost” the subject and that would ultimately need to be replenished during the recovery period.

The aim from this 20 minute performance test was to calculate the mean power output that each subject could sustain for the duration of each 20 minute cycle period. In order to assess the physiological intensity at which the cyclists were performing this test, data was compared to data from each individual cyclist’s response to the maximal exertion trial. By optimising the intensity at which the cyclists were pacing these trials, it was possible to estimate the mechanical force needed for each 20 minute bout of exercise, and hence develop an individual pacing strategy for each cyclist. It also allowed the development of strategies for worst case scenarios where a cyclist would be forced to cycle further than the normal 20 minute rotation. By establishing how long a cyclist could cycle before total failure, and recovery, at a given intensity it would benefit the team in making decisions on whom to send out onto the road next.

As one of the aims for the study was measurement of total calorific expenditure it was important to know the relative effort produced by each of the participants during a cycling period. It is true that the effort per participant will differ due to terrain, weather, hydration and many other variables for each of the cycling periods. However, by attempting to develop a course that mimicked the overall course structure it was a step towards getting a picture of what occurred during the race.

## 4. Sensors Used in the RAI

Given the nature of this project a number of measurement techniques were deployed to quantify a broad range of aspects that may indicate the impact of ultra endurance cycling, as detailed in Table 3. This section is split into two broad categories, namely *biological sensing* in which data cannot be extracted simply and quickly, and *physical sensing* where instantaneous data can be simply extracted however, this data contains greater noise. Given exposure to random weather conditions, perspiration deposits, competitive environments, and the length of the event, noisy data capture and sensor failure was unfortunately inevitable. To compensate for this a wide range of complementary physical sensors such as: accelerometers in multiple locations; GPS; respiration monitoring; heart rate monitoring; power output sensors; and video capture, were used at various stages during the race. The addition of a follow vehicle behind the cyclists presented a chance to locate sensors within the follow vehicle to monitor the cyclists at all times from an external aspect.

### 4.1. Biological Sensing

In order to assess the physiological and biochemical changes occurring during the event a variety of different monitoring techniques were used.

#### Haematological Analysis

Blood was drawn from the anticubital vein in participants’ forearm. Samples were drawn as both whole blood and as blood stored in an anti coagulant EDTA. A complete blood count was performed on the EDTA blood sample for the measurement of red and white blood cells, haematocrit, haemoglobin, and several other blood metabolites. This allowed for the separation of both blood plasma and serum after the samples were centrifuged at 1,500 rpm for 15 minutes at 4 degrees Celsius. Blood plasma and serum were stored at −80 degrees Celsius for further analysis post event. Post race serum and plasma analysis is in progress and due to focus on several blood metabolites including; Creatine kinase, lactate dehydrogenase, myoglobin, and IgA.

#### Saliva Analysis

Saliva was sampled below the front of the tongue after rinsing the mouth on an absorbent sorbet. This allowed for a sample to be gathered from a standard site during all points in the race for the assessment of hormonal changes. The hormones that were included in the study were; cortisol, testosterone, secreatory IgA, and C-reactive protein. After samples were frozen they were centrifuged to remove the saliva for assay post event.

#### Heart Rate Analysis

Heart rate was continually measured during trials using a Polar heart rate monitor which sampled every 5 seconds. This allowed for accurate assessment of cardiovascular response to exercise during the 20 minute performance trial and further development of training strategies for the cyclists post testing.

#### Respiration Analysis

Respiration was monitored during maximal exercise trials using a metabolic cart, Innocor*™* (Innocor, Denmark). This allowed for breath-by-breath measurement of inspired and expired carbon dioxide and oxygen through inbuilt sensors. During the race itself respiration was monitored for a period on participants using a wearable sensors vest developed by QinetiQ (formerly Foster Miller) which allows for measurement of respiration via an expandable array of sensors. These sensors measure the rate and amount of expansion for the chest cavity and translate this into a measure for respiration rate. This combined with a built in EKG accurate heart rate monitor, thermistor, and GPS module allow for a high level of portable, real-time transmitted, sensor measurement of exercise activity.

### 4.2. Physical Sensing

A range of physical sensing devices were deployed during the event, namely camera, GPS location loggers, accelerometers mounted on the frame of each participant’s bicycle, and also accelerometers mounted on the right ankle of each cyclist.

#### Location Analysis

**Figure 2.**
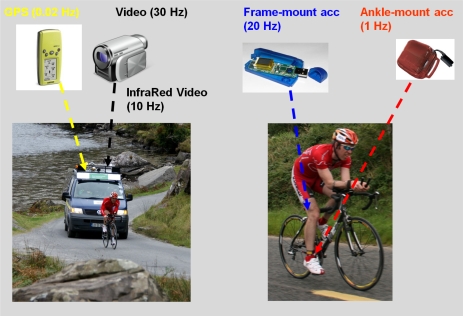
Overview of Physical Sensing Devices Used.

Figure 2 provides an overview of the physical sensing devices used throughout the entire duration of the cycle race. GPS location data was captured at a rate of once per minute throughout the race. The elevation element of this dataset was used to model the difficulty of the course due to resistance offered by inclines,. Despite some capture difficulties, 4,556 GPS readings were captured in the follow van used throughout the race.

#### Video Analysis

Video footage was captured so as to support post-race data analysis, particularly to review abnormalities as suggested by data captured from the sensors. Video was captured from the follow van and was either visible spectrum video (daytime) or video from an infra-red camera with infra-red floodlights mounted on the roof of the follow van for night-time cycling. In total approximately 22 hours of video footage, totalling 16.4 GB of data was captured.

#### Bike Motion Analysis

A frame mounted accelerometer, a Gulf Coast Data Concepts X6-2A, captured and stored x, y, & z axes data at a rate of 20 Hz. By mounting this accelerometer on the frame of the bike was to detect if the bike is active or not, and also for possibly detecting whether the bike may be cornering or not. Unfortunately due to the harsh environment in which the race operates, this sensor was unable to record data at some stages through the race for all of the participants. In total 21,161,402 data readings were captured through the race.

#### Cyclist Ankle Motion Analysis

An Actigraph GT3x ankle mounted accelerometer sampled movement data (acceleration due to gravity along three separate axis) every second. Data that was sampled every second was a sum total of the number of “counts” captured at a rate of 33 Hz on the x, y, and z axes. By mounting these accelerometers on the ankles of the participants for the entire duration of the race it was possible to collect data on how active they were throughout the entire duration of the event. The ankle clasp proved to be an unobtrusive place to mount the accelerometer. In total 4,081,267 data readings were captured through the race.

## 5. Sensor Data Processing

One of the major goals of this study was to identify automated techniques that can provide an *estimation* of the energy expenditure of athletes during both cycling and rest periods in the race. To investigate this, attention was focused on the ankle mounted accelerometers(Unfortunately the frame mounted accelerometers experienced failure at various stages during the race due to a lack of robustness in dealing with this inherent noisy environment), which successfully recorded data at a granularity of 1 second throughout the entire race for all participants.

In order to use this data, a definition for a cycling event detection model needed to be developed. Thus: A cycling event refers to; a period of time where the participant was actively engaged in cycling, as opposed to non-cycling events where the participant was not cycling. Sensing these events provide a difficult challenge as at various times the participants could be quite active during non-cycle periods (e.g., walking or even jogging before stretching muscles, etc.). To identify cycle events, data was processed as follows:

1. Raw Actigraph “activity count” data is gathered every second, giving a magnitude value for total acceleration on x+y+z axes.
2. This data was summated for every minute of the race, which removes a lot of the excess noise.
3. The application of a smoothing window of 10 minutes over all this data. Median smoothing was used.
4. 128I dentification of time blocks when activity levels were above 40% (or detected maximum of 1 minute blocks identified in step 2), minimum length of cycle period must be 10 minutes (to eliminate noise). Once the cycle periods were detected, it was assumed that all other periods of time were rest periods.
5. For each period, calculate the minimum/maximum/median/mean values of associated activity count.

**Figure 3.**
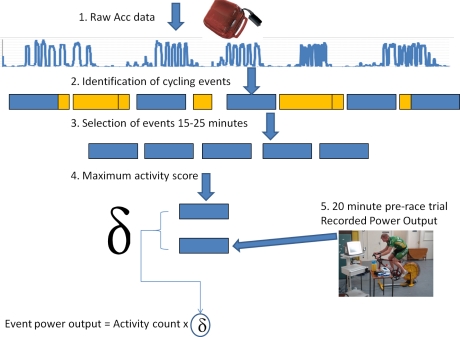
Overview of the process to estimate energy expenditure.

Following the data collection the next stage was to then estimate the energy expenditure of each of these events. This process is illustrated in Figure 3. Once the cycle periods were detected, it was assumed that all other periods of time were rest periods. For each of the periods of activity it was then considered the median effort score throughout the period. Thereafter only the events that were 20 ± 5 minutes in duration were considered as it is quite likely that the effort expended during those events would mirror that of the 20 minute pre-race trial (discussed in Section 3.1). From these filtered events for each participant the event with the maximum recorded mean activity score was selected, and correlated to the average power output recorded in the pre-race trials. Using this correlation index, it was possible to apply a multiplication factor to the mean activity score of all the other events to estimate the power output of the cyclists. The next section describes the power output, and as Watts can be converted to kilocalories, how an inference can be performed on the calorific expenditures exerted by the participants through various stages of the race.

It is understood that a number of inferences (and thus potential inaccuracies) are introduced in this process. However, there is merit in producing estimations of the energy expenditure of athletes in ultra-endurance races. An area of future work is in carrying out experiments in controlled environments with the ankle-mounted accelerometers validated against gold standard power meters and is currently underway.

### 5.1. Custom User Interface to Review Multimodal Data

**Figure 4.**
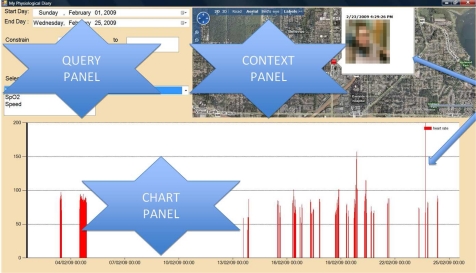
Interface to assist in reviewing multimodal data captured during the race.

To review the various sources of captured data a system was built which exploits certain characteristics of the human memory system to help contextualise and validate the occurrence of given events. To achieve this needed the development of the interface in Figure 4 containing a query panel, a chart panel, and a context panel.

The query panel allows for adaptive searching for data based on time predominantly, thus exploiting the fact that humans have an acceptable memory of when events of interest temporally occur [17]. The chart panel provides a visual representation of the physiological activities, thus allowing immediate identification of distinct events (potential anomalies) e.g. when there was a sudden change in activity against the normal pattern (e.g., a wrong direction was taken and the cyclist had to turn back). By clicking on any value of interest, the user can shift attention to the context panel. This panel is very important in providing other contextual cues to the user to help provide additional memory cues to better explain the reasons for a certain physiological reading. Contextual cues can include the location of the user, and also any video that may have been captured at that time.

**Figure 5.**
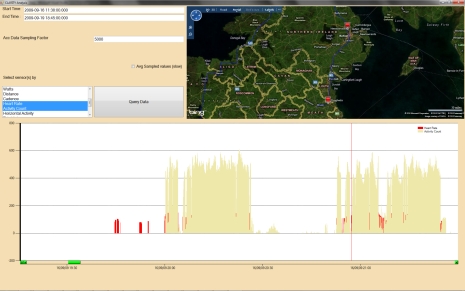
“My Physiological Diary” — identify daily trends across multiple data sources.

As can be seen in Figure 5 multiple (Min-Max normalised [18]) physiological values can be charted at the same time to visually inspect for any relationships or correlations among the values. Using the query panel, it is possible to “group graphs by” each day, thus helping to quickly identify patterns in the data.

## 6. Preliminary Results from Data Analysis

Initial investigation of complete blood count (CBC) data showed highly significant differences for white blood cells (WBC) and granulocyte count (*p* < 0.01), and significant differences for haematocrit, hemoglobin, lymphocytes, and red blood cells (*p* < 0.05). No significant difference was observed for changes in body mass or urine specific gravity during the study leading to a belief that plasma volume remained stable for all samples, however correction for changes has yet to be undertaken. Post race serum and plasma analysis show changes in metabolites during the RAI compared to baseline and an eventual return to basal levels over a two week period post-race, highlighting the stresses placed on the participants in this ultra endurance event.

**Figure 6.**
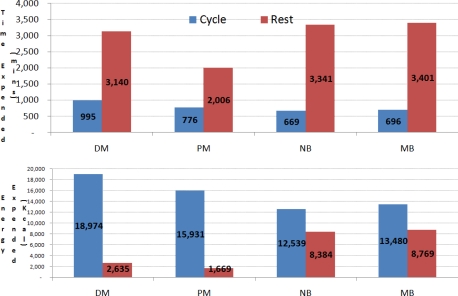
Time (above) and energy (below) expended by each cyclist over the entire race.

Estimated total time and energy expenditure (while cycling/resting) was automatically calculated from the ankle mounted accelerometers and is presented in Figure 6 for each individual member of the team. The average cycle period across the 4 participants was 22 minutes (with 426 kcal expended), and the average rest period was 90 minutes (with 177 kcal expended), however this would be confounded by longer breaks of 4 and 6 hours during the race.

**Figure 7.**
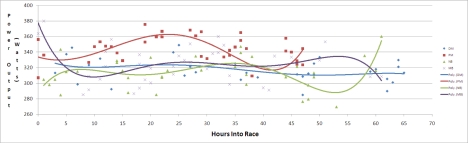
Power output of each cycling period as race progressed.

Furthermore of the 145 detected cycling periods across the 4 participants, it was possible to investigate the duration of each cycling period at the race progressed. It was seen that all four cyclists had longer cycling periods in the first 15 hours of the race, but thereafter possibly due to the cumulative effects of fatigue, the cycling periods became shorter in duration, with the exception of subject PM who had to cycle for unusually long periods approximately 40 hours into the race due to a tactical error by the team. In the last 10 hours of the race subject DM had cycling periods of longer duration due to being a well-trained endurance cyclist and recovering better from each cycling period. Estimated power output was calculated for each cyclist throughout the race from the ankle mounted accelerometers. As the race progressed, large fluctuations in power output were observed for each cycling period as presented in Figure 7.

**Figure 8.**
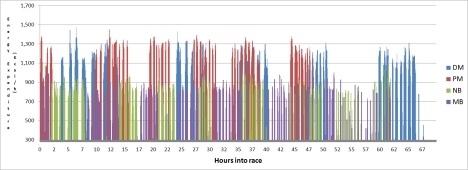
Calorific expenditure of each cycle period through the race.

An overview of the calorific expenditure for each of the cyclists throughout the race, period by period, is presented in Figure 8.

## 7. Discussion of Results from Data Analysis

In order to assess the impact of the race on each of the cyclists it was necessary to view their data in both an individual and a group format. By initially looking at what happened to each cyclist during the race, the authors aimed to gain a greater understanding of how the team performed during the race, how this resulted in their final position, and what could be done in future studies.

### 7.1. Individual Action Recognition

By viewing each of the cyclist’s instances above the activity count threshold (Threshold was parameterised using mean thresholding, and is an area for future optimisation), it became possible to establish the major activity periods of each cyclist. These major activity periods related to the times that the cyclists were actively cycling. Each cyclist was working in a pair, DM and NB; MB and PM, alternating cycling bouts for short periods. This allowed the rest periods to be highlighted, most of which fell inline with the team 128s planned race strategy.

Figure 8 shows the 64 hours of race time, when all periods of activity are layered on top of each other for each cyclist. Generally there is at least one cyclist cycling at any time. There were cycling periods missed by the cycle detection algorithm, most likely due to the noisy data capture environment. By further refining the algorithm it would be possible to minimise this loss.

**Figure 9.**
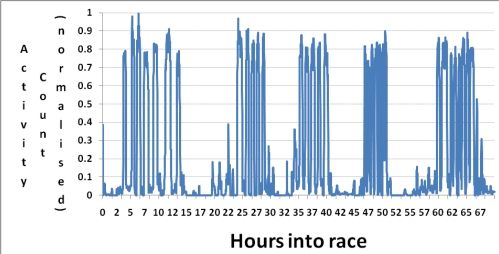
Raw activity (smoothed over one minute window) for subject DM.

Figure 9 is a case study on one of the cyclists (DM) and shows the 5 main activity periods during the entire race. This graph is taken from the raw data (smoothed over one minute window) and plotted against the time that it took the team to complete the race.

Unfortunately full data sets were not recorded for all participants. NB and MB: the GTX3 ankle mounted accelerometers failed during the final few hours. PM: unfortunately there was also a sensor failure for the final 20 hours.

**Figure 10.**
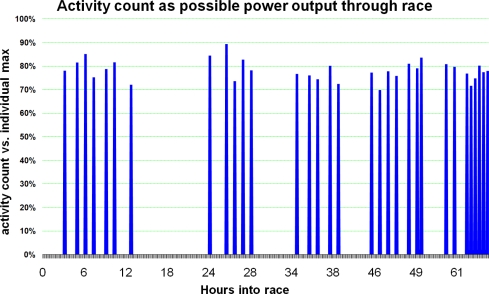
Activity count (relative to maximum effort through race) for subject DM.

To validate the output from the ankle mounted accelerometers results focus on data from on subject DM. Figure 10 illustrates that our cycling event detection algorithm can identify the major cycling events during the race are visible. Figure 10 shows the points at which the subject was exercising above a certain activity threshold, *i.e.*, 40% of their maximum over a median smoothed 10 minute time window, which are recognised as either cycle or rest events as described by the algorithm in Section 5. However, with sensor failure and noisy readings being inevitable in this environment, there were periods of dead time where not all data was recognised. Unfortunately it was impossible to quantify the amount of time, both cycling and resting, lost during the race, and hence it is quite clear that the outputs referred to in this article are estimated or inferred values. However as will become evident the power output and energy expenditure values are highly promising.

### 7.2. Recovery Period

**Figure 11.**
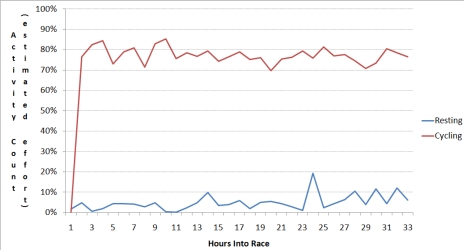
Subject DM rest *vs.* cycle.

By taking the calculated outputs from the cycle event detection algorithm, it is possible to estimate the activity that the cyclists were undergoing during rest periods, see Figure 11. The rest and cycling curves take into account both ride and rest periods and give an overall running average of both.

### 7.3. Estimated Power Output

**Figure 12.**
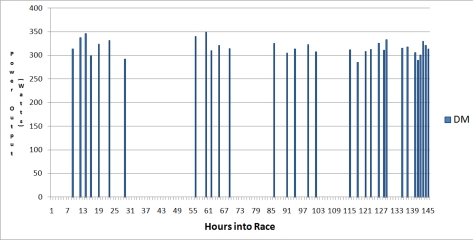
Subject DM power output through race.

The ability to quantify the mechanical power generated by a cyclist over a period of time is one of the few absolute measures of cycling intensity available to cyclists and coaches today. By combining the previous 20 minute time trial data gained during preliminary physiological testing (see Section 3.1), and the activity count data gained during the race (through considering events 20 ± 5 minutes in duration) as described earlier in Section 5, an estimated power output for each cycle period could be calculated. This estimated power output is represented in Figure 12.

**Figure 13.**
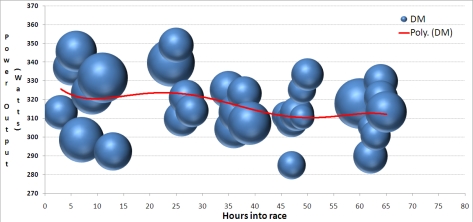
Progression of cycling periods for subject DM through race. X-axis is hours into race, y-axis indicates average power output for each period, and size of bubble indicates duration of period.

In Figure 13 it can be seen that the power output generated throughout the race (*i.e.*, position of bubble on y-axis), and also for how long subject DM was producing this power output (*i.e.*, the duration of the period, where bubble radius is proportional to the number of minutes of the individual cycle period). It is evident that periods were longer at the start of the race, with periods at their shortest around 50 hours into the race, but with longer periods again in the last 5 hours.

### 7.4. Estimated Energy Expenditure

After establishing that the power output from each cycling period made was within reported values, further investigation into the calorific expenditure of each cyclist was undertaken. As expected energy expenditure during the race fluctuated for each cyclist. This was due to many factors from varying terrain, which cause the cyclists to adopt a more, or less, aggressive manner of cycling. Weather conditions such as wind which would result in participants having to generate more power to overcome headwinds. Increased fatigue over the duration of the event would naturally be another significant factor.

**Figure 14.**
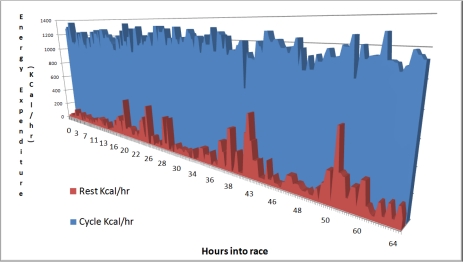
Energy expenditure for cycle and rest events across all four participants in the race. Energy not conserved as well towards middle and end of the race as at the start.

**Figure 15.**
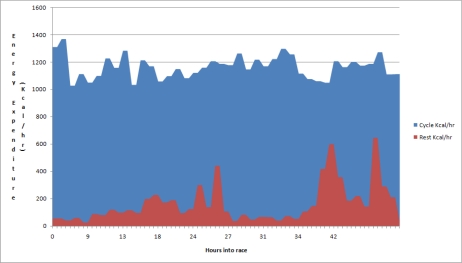
Subject DM energy expenditure through race.

An interesting result was that as the race progressed the participants became less efficient at recovering between cycle bouts. As Figures 14 and 15 show, the level of energy expenditure during rest periods actually increased across the 4 participants while nearing the end of the race. These periods of increased energy expenditure during rest would act so as to detrimentally affect the race performance of cyclists as they would have unnecessarily expended energy during recovery. This was commented on by the cyclists themselves who felt restless during the latter parts of the race, and felt as though they needed to pick up some of the support work that, in their eyes, was not being performed by the race crew. Future recommendations in this area would include developing a mechanism of feedback to the cyclists themselves so as to remind them to rest and expend less energy, rather than having to rely on the race crew to tell them to do so, who in the latter parts of the race would also have been suffering from fatigue.

By examining the data post-race, a greater understanding of how the energy demands of the event changed as the race progressed. By layering this data over an analysis of the course from either GPS or altimeter data it would be possible to get a greater understanding of the needs of long distance cycling for future events. However, in order to make this data more relevant to cyclists and their coaches it may necessitate the introduction of other sensing modalities such as real time heart rate monitoring, and wearable respiration sensors, etc. Another key factor that was not assessed during the event was any sensing of the physical environment. As weather can play a large part on cycling, and its affect on the cyclist, it stands to reason that sensing of the environmental conditions during the race could lead to a greater understanding of their effects on the participants. All these technological challenges lie ahead, but the authors believe this work is a valuable initial analysis into the direction for future research into the role sensor data processing can play in assisting ultra endurance cyclists.

## 8. Conclusions

In this work a new testing bed for sensing devices was introduced, namely competitive ultra-endurance cycling events. The physiological costs these events place on cyclists, and the difficulties in reliably capturing data within these environments, make it an interesting area for study. Previous measurement of energy expenditure during such cycling events necessitated the use of expensive power monitoring devices, or obtrusive heart rate based monitoring. In this work the potential of deploying a cheap simple ankle mounted tri-axial accelerometer as a means of estimating the energy expenditure of cyclists was introduced. An algorithm was introduced to process the raw motion data stream to identify events when the participants were cycling/resting. From this it is possible to estimate the energy expended while cycling/resting through the race.

From this automated process of semantically enriching the raw data into meaningful information, investigators were able to identify some of the demands placed on ultra endurance cyclists. During the start of the race periods of cycling were both longer and completed at a higher average power output, whereas cycling periods were shorter and less intense as they progressed into the middle parts of the event. Towards the latter parts of the race there was an increase in power output (but not cycling length), illustrating a change in the ability of the cyclists to work harder when the final goal was in sight, mentally overcoming any physiological fatigue. Encouragingly this was complemented by traditional analysis of metabolic samples, and psychological function tests, in an attempt to measure this change.

To further evaluate these techniques would necessitate a greater number of participants and correlation with on-bike power meters in order to assess the accuracy of the developed algorithm. However this work has shown a new technique to estimate both the power output and calorific expenditure of cyclists in a cheap, reliable and simple manner in ultra endurance cycling events.

This paper deliberately does not focus on the results or analysis of the physiological performance during the race, but highlights the range of sensors that were used in the RAI By using a wide and diverse range of sensors it was possible to generate large amounts of information to help with the analysis of the RAI. At the moment there is a distinct lack of information of the needs, and the effects, of ultra endurance cycling Through sensors based studies such as this it may be possible to add to the body of literature and provide important information to the coaches, and cyclists themselves.

## Figures and Tables

**Table 1.** Profile Of Athletes in this Study.

ID	Age (yrs)	VO_2 *max*_ (*ml / kg / min*)	VO_2 *max*_ (*L / min*)	HR*_max_* (*bpm*)	Power*_max_* (W)	Lactate*_max_* (*mmol*)	Pwr @ LT (W)	HR @ LT (*bpm*)

DM	39	71.7	5.3	171	400	14.1	330	152
PM	36	76.4	6.3	174	425	10.8	331.3	147.9
NB	51	60.5	4.8	179	375	12.7	285	152.6
MB	43	66	5.3	182	375	8.2	327	170

**Table 2.** Athlete Performance in Pre-Race Trial.

ID	Distance (km)	Power Output*_Avg_* (W)	Power Output*_Avg_* (W/kg)	HR*_Avg_* (*bpm*^−1^)	HR*_Max_* (*bpm*^−1^)	Cadence (RPM)	Speed*_Avg_* (km/h)

DM	11.14	328.05	4.48	155.2	172.5	132.8	33.39
PM	11.48	358.9	4.34	124.5	168	115.15	34.44
NB	10.08	277.75	3.59	157.25	175	146.65	30.26
MB	10.02	265.3	3.5	154.2	192.5	135.45	30.9

**Table 3.** Data Collected During Race.

Information Source	Num Data Readings Collected in Race	Data Frequency

Ankle Mount Accelerometer	4,081,267	1 Hz
Frame Mount Accelerometer	21,161,402	20 Hz
GPS	4,556	0.1 Hz
Video	16.4 Gb	25 fps
Heart Rate / Respiration Vest	2,303	Breath-by-breath
Blood samples	72 vacutainers (864 mL)	Every 24 hours in race

**Table 4.** Time Spent Cycling/Resting as Estimated by Cycle Event Detection Algorithm.

Subject	Cycling minutes	Resting minutes

DM	995	3140
PM^1^	776	2006
NB^2^	669	3341
MB^3^	696	3401

Total	3,136 (52h 16m)	11888
